# Preoperative Vaginal Microbiota and Risk of Complications After Transobturator Tape Surgery: A Pilot Study

**DOI:** 10.3390/jcm15124540

**Published:** 2026-06-11

**Authors:** Karolina Chmaj-Wierzchowska, Kinga Bednarek, Beata Serbin, Jowita Staszewska, Julian Graś, Kacper Żmuda, Aleksandra Makarewicz, Olaf Zamojcin, Adrian Mruczyński, Maciej Wilczak

**Affiliations:** 1 Department of Maternal and Child Health and Minimally Invasive Surgery, Poznan University of Medical Sciences, 60-701 Poznan, Poland; 2 Department of Advanced Interventional Therapies in Gynecology and Urogynecology, Poznan University of Medical Sciences, 60-701 Poznan, Poland; 3 Student of the Faculty of Medicine, Poznan University of Medical Sciences, 60-701 Poznan, Poland93441@student.ump.edu.pl (J.S.); 93270@student.ump.edu.pl (J.G.); 93481@student.ump.edu.pl (K.Ż.); 94148@student.ump.edu.pl (O.Z.)

**Keywords:** stress urinary incontinence, transobturator tape, vaginal microbiota, dysbiosis, *Lactobacillus*, postoperative complications, urogynecology

## Abstract

**Background:** The vaginal microbiota plays a crucial role in maintaining urogenital health, and its disruption may contribute to adverse surgical outcomes. Transobturator tape (TOT) procedures are widely used in the treatment of stress urinary incontinence (SUI), yet factors influencing postoperative complications remain incompletely understood. This study aimed to evaluate the association between preoperative vaginal microbiota and postoperative complications following TOT surgery. **Methods:** This pilot observational study included 24 women diagnosed with SUI who underwent TOT surgery between February and December 2023. Prior to surgery, all patients received standardized preoperative preparation, including local estrogen therapy and antimicrobial treatment. Vaginal swabs were collected and analyzed using quantitative culture methods to assess microbial composition. Clinical, microbiological, and surgical data were analyzed. Associations between microbiota profiles and postoperative complications were evaluated using univariate and multivariable statistical analyses. **Results:** Postoperative complications occurred in 5 patients (20.8%). Patients with complications had significantly higher body mass index (BMI) (33.8 ± 5.0 vs. 27.9 ± 5.1 kg/m^2^; *p* = 0.028) and a higher prevalence of previous surgeries (80% vs. 42%; *p* = 0.048). Microbiological analysis revealed that *Lactobacillus* spp. deficiency was more frequent in patients with complications (60% vs. 21%; *p* = 0.048), as was the presence of mixed microbial flora indicative of dysbiosis (100% vs. 47%; *p* = 0.041). Multivariable logistic regression identified BMI (OR = 1.16; *p* = 0.031), previous surgeries (OR = 3.9; *p* = 0.049), *Lactobacillus* spp. deficiency (OR = 4.8; *p* = 0.038), and mixed flora (OR = 6.2; *p* = 0.027) as independent factors associated with postoperative complications. **Conclusions:** Preoperative vaginal microbiota may significantly influence outcomes following TOT surgery. Vaginal dysbiosis, particularly *Lactobacillus* spp. deficiency and mixed microbial flora, appears to be associated with an increased risk of postoperative complications. These findings suggest that microbiota assessment could be considered in preoperative evaluation. Further large-scale studies using advanced microbiome analysis are warranted to confirm these results.

## 1. Introduction

The vaginal microbiota is a complex and dynamic ecosystem whose balance is crucial for women’s reproductive health [1,2,3]. Its functionality is intricately linked with hormonal regulation, particularly estrogen levels and glycogen availability. Estrogens promote epithelial proliferation and the accumulation of glycogen, which serves as the primary energy source for bacterial species such as *Lactobacillus*. These microorganisms produce lactic acid, thereby facilitating the maintenance of an acidic pH and inhibition of the growth of pathogenic bacterial species. During menopause, estrogen deficiency induces substantial alterations, including epithelial thinning, reduction in glycogen reserves, and loss of *Lactobacillus* prevalence, resulting in an elevated pH level and the development of dysbiosis, which has considerable clinical implications, manifesting as symptoms of urogenital atrophy [4,5,6,7].

Local estrogen therapy remains the cornerstone approach to address these alterations; this therapy stimulates epithelial regeneration, increases the glycogen level, and promotes the restoration of a healthy physiological microbiota. These positive changes normalize the pH level and enhance the protective barrier function. During preparation for urogynecological procedures, including the treatment of stress urinary incontinence (SUI) through the transobturator tape (TOT) technique, this therapy improves tissue elasticity and vascularization, thereby reducing the risk of complications such as implant erosion. SUI poses a considerable health risk, affecting 25–45% of women, particularly during the perimenopausal period. It is defined as the involuntary leakage of urine following an increase in intra-abdominal pressure without concurrent detrusor muscle activity [8,9]. SUI has multifactorial etiology, including prior childbirth, obesity, chronic cough–related conditions, pelvic surgeries, and age (9–14 years). Estrogen deficiency further impairs the function of the urethral support apparatus by altering vascularization and collagen metabolism [10,11,12,13,14,15].

In surgical management, minimally invasive procedures using mid-urethral slings are considered the gold standard. The transobturator tape (TOT) technique reduces the risk of complications associated with the retropubic approach while simultaneously maintaining high efficacy [16,17]. In postmenopausal patients exhibiting features of urogenital atrophy, local estrogen therapy plays a pivotal role in preoperative preparation. This intervention improves tissue quality, facilitates surgical dissection, and supports implant integration. It also promotes the restoration of the vaginal microbiota by re-establishing *Lactobacillus* prevalence and lowering the pH value, which is critical for stabilizing the perioperative environment [18].

The vaginal microbiota plays a crucial role in maintaining urogenital health, and its disruption may contribute to adverse surgical outcomes. Transobturator tape (TOT) procedures are widely used in the treatment of stress urinary incontinence (SUI), yet factors influencing postoperative complications remain incompletely understood. This study aimed to evaluate the association between preoperative vaginal microbiota and the risk of complications following TOT surgery.

## 2. Materials and Methods

### 2.1. Study Group

The study group comprised 24 women diagnosed with SUI, who were hospitalized at the Department of Maternal and Child Health and Minimally Invasive Surgery of the Gynecology and Obstetrics Clinical Hospital of Poznan University between February 2023 and December 2023. The criterion for inclusion was a confirmed diagnosis of SUI in accordance with the current clinical guidelines [2]. After obtaining informed consent, cervical canal swabs were collected from the participants before implementing the preoperative preparation protocol preceding surgical treatment. The samples were obtained for quantitative microbiological culture. All surgical procedures were performed by experienced urogynecological surgeons specialized in female pelvic floor surgery (approximately 140 TOT procedures are performed annually). Standardized operative techniques and perioperative protocols were applied in all cases. Following surgery, patients remained under systematic follow-up, including the assessment of postoperative complications and other clinical parameters, conducted until December 2025, with scheduled follow-up visits at 3, 6, and 12 months after the procedure.

### 2.2. Preoperative Preparation Protocol in Urogynecology

All patients underwent a standardized preoperative preparation protocol for improving tissue trophicity, restoring the physiological vaginal microbiota, and reducing colonization by potentially pathogenic microorganisms. As an integral component of this preparation, local estrogen therapy was administered using vaginal suppositories containing 0.5 mg of estriol, applied once daily at bedtime for 14 days, followed by a maintenance regimen comprising twice-weekly administration for an additional 28 days. Adjunctive antimicrobial therapy was administered through vaginal tablets containing 500 mg of nifuratel (Nifuratelum) and 200,000 IU of nystatin (Nystatinum); these tablets were prescribed once daily at bedtime for 12 consecutive days, commencing 12 days prior to the planned surgical treatment. The applied protocol was empirical in nature and was routinely implemented as part of preoperative preparation. All patients completed the full preoperative preparation regimen in accordance with the recommendations, with no reported deviations. The adopted preparation strategy was designed to optimize wound healing conditions, improve tissue quality, and potentially reduce the risk of complications following mid-urethral sling implantation.

### 2.3. Chemicals and Media Used

Amies transport medium (for quantitative microbiological culture). AmpliSens^®^ DNA-sorb-AM (for bacterial DNA isolation; Ecoli Dx, Prague, Czech Republic)). Microbial tests were conducted using the following media: Transparent Chromogenic UTI Medium (OXOID), Columbia Agar with Sheep Blood (OXOID), Sabouraud Glucose Selective Agar (OXOID), Schaedler Anaerobe KV Selective Agar (OXOID), *Gardnerella vaginalis* selective medium (OXOID), Rogosa + H_2_O_2_ Agar (HEIPHA), MacConkey No. 3 (OXOID), Enterococcus Agar (Bile Esculin Azide Agar, GRASO), and Chromogenic *Candida* Selective Agar (GRASO). The bacterial species were identified based on colony morphology, Gram staining features, and biochemical characteristics.

### 2.4. Quantitative Microbial Culture

The swabs were collected for quantitative microbial culture (using Amies transport medium). Within 48 h of collection, the swabs were delivered to the Microbiology Laboratory of the Institute of Microecology in Poznan (Sielska Street) for analysis. Microbial analysis involved quantitative culture on preselected selective and differential media, following the Standard Operating Procedure developed by the Institute of Microecology. The culture process identified bacterial and fungal species (CFU/mL), including *Streptococcus agalactiae* (group B beta-hemolytic streptococcus), *Gardnerella vaginalis*, anaerobic bacteria, other potentially pathogenic bacteria unique to each patient, *Lactobacillus* spp., *Lactobacillus* spp. producing H_2_O_2_, and yeast-like fungi of the genus *Candida*.

### 2.5. Statistical Analysis

Data analysis was performed using Statistica (Cloud Software Group, Inc., Fort Lauderdale, FL, USA) (2023), Data Science Workbench (version 14), The Jamovi Project (2022) Jamovi (version 2.3) [Computer Software], and Microsoft Excel (Microsoft Office (2019), version 2205)). For comparison of patients with and without complications in terms of age, body mass index (BMI), number of deliveries, and duration of previous surgeries, data are expressed as mean ± standard deviation (SD) or median (interquartile range, IQR). Student’s *t*-test or Mann–Whitney *U* test was used for comparing continuous variables, while the χ^2^ test was applied for comparing categorical variables. To assess the relationship between vaginal microbial profile and the occurrence of complications following TOT surgery, data are expressed as counts and percentages (%). The χ^2^ test or Fisher’s exact test (for small sample sizes) was used for analysis. A *p*-value of <0.05 was considered statistically significant.

### 2.6. GenAI

During the preparation of this manuscript/study, the author(s) used [ChatGPT (OpenAI, GPT-5.3, accessed April 2026)] for the purposes of language editing and improvement of readability, paraphrasing and refining the wording of the manuscript, generating a figure based on the authors’ input and specifications. The authors have reviewed and edited the output and take full responsibility for the content of this publication.

## 3. Results

### 3.1. Clinical Analysis

The analysis included 24 patients aged 29 to 72 years (mean 51.8 ± 10.6 years). The mean BMI was 29.7 ± 5.8 kg/m^2^, with most patients classified as overweight or obese. The median number of deliveries was 2. The procedure of TOT was most frequently performed using the outside-in technique (54%) and less commonly using the inside-out technique (38%). The mean operative time was 26.8 ± 5.4 min. The mean urethral length measured by ultrasound was 32.1 ± 3.6 mm. Table 1 shows the characteristics of the study group undergoing TOT surgery, including demographic and clinical parameters as well as operative time.

In the analyzed group, complications occurred in 5 patients (20.8%). As shown in Table 2, most complications were related to vaginal mesh exposure requiring surgical intervention.

Patients who developed complications had a significantly higher BMI compared to those without complications (Table 2, Table 3 and Table 4). BMI exhibited a significant association with the risk of complications (*p* = 0.028).

### 3.2. Analysis of Vaginal Microflora

In the studied group of 24 women aged 29–72 years (BMI 21.45–44 kg/m^2^), the vaginal microbiota was quantitatively assessed in relation to reference values (RV) (Table 3). The abundance of hydrogen peroxide (H_2_O_2_)-producing *Lactobacillus* spp. ranged from below the detection threshold (↓RV) to 1 × 10^9^ CFU/mL. In approximately half of the cases, the values reached the range of ≥10^7^–10^9^ CFU/mL, corresponding to reference levels, whereas a reduced abundance below RV was observed in the remaining patients.

A similar distribution was observed for the total *Lactobacillus* spp. count, which ranged from below the detection threshold (↓RV) to 1 × 10^9^ CFU/mL, with a subset of women demonstrating a marked depletion of these bacteria. Among aerobic and facultative anaerobic potentially pathogenic bacteria, *Enterococcus* spp. were most frequently isolated, with counts ranging from 2 × 10^4^ to 1 × 10^8^ CFU/mL. High concentrations of *Enterococcus* spp. (>10^6^ CFU/mL) were observed in multiple cases, particularly in association with reduced levels of *Lactobacillus* spp. *Escherichia coli* was detected in the range of 3 × 10^7^ to 1 × 10^9^ CFU/mL, with values ≥ 10^8^ CFU/mL recorded in several samples, suggesting substantial colonization. Coagulase-negative staphylococci (CNS) were also identified. The concentrations of CNS ranged from 2 × 10^4^ to 2 × 10^7^ CFU/mL, while *Staphylococcus aureus* (2 × 10^5^ CFU/mL) was identified in single cases. In some samples, other Gram-negative bacteria were detected, including *Klebsiella oxytoca* (4 × 10^4^–4 × 10^8^ CFU/mL), *Enterobacter* spp. (2 × 10^6^ CFU/mL), and *Morganella morganii* (6 × 10^6^ CFU/mL), indicating substantial diversity of opportunistic microbiota. Fungi of the genus *Candida* were identified in multiple cases. The concentrations of *Candida albicans* ranged from 5 × 10^6^ to 3 × 10^7^ CFU/mL, whereas the concentration of *Candida lusitaniae* was approximately 5 × 10^7^ CFU/mL. These values exceed levels typically associated with physiological colonization, suggesting potential clinical relevance. Selected cases showed the presence of anaerobic bacteria, such as *Prevotella bivia*, at concentrations of approximately 2 × 10^8^ CFU/mL, which may indicate microbial imbalance commonly observed in bacterial vaginosis. In most samples, *G. vaginalis* and other anaerobic bacteria were present below RVs. The analysis revealed considerable variability in the abundance of both protective bacteria and opportunistic microorganisms (Table 4).

Patients with complications exhibited a significantly higher incidence of *Lactobacillus* spp. deficiency (60% vs. 21%; *p* = 0.048) and a higher prevalence of mixed flora (100% vs. 47%; *p* = 0.041) compared to patients with no complications. However, both these groups showed no significant differences in the incidence of individual pathogens, including *E. coli*, *Enterococcus* spp., and *Candida* spp. Table 5 shows the relationship between vaginal microbial profile and the occurrence of complications following TOT surgery.

### 3.3. Complication Analysis

**Figure 1:**
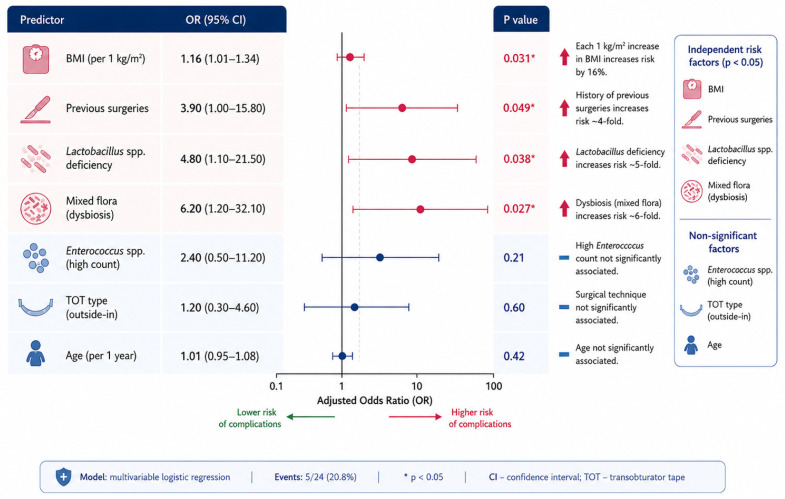
Multivariable risk model for postoperative complications after transobturator tape (TOT) surgery.

A multivariable logistic regression analysis was conducted to identify independent risk factors associated with complications following TOT surgery (Table 6 and Figure 1). The model included variables with clinical relevance and those showing a significant association in univariate analysis. A *p*-value of <0.05 was considered statistically significant. The multivariable logistic regression analysis revealed the association of several independent risk factors with postoperative complications after TOT surgery. The results indicated a significant effect for higher BMI (OR = 1.16; *p* = 0.031) and a positive surgical history (OR = 3.9; *p* = 0.049). Among the microbial factors examined, *Lactobacillus* spp. deficiency (OR = 4.8; *p* = 0.038) and the presence of mixed flora indicative of dysbiosis (OR = 6.2; *p* = 0.027) were significant. In contrast, patient age, the type of surgical technique used (inside-out vs. outside-in), and the presence of *Enterococcus* spp. showed no significant effects on the risk of complications. The results obtained from the logistic regression model warrant careful interpretation because of the small sample size and the limited number of events. The presence of wide confidence intervals indicates limited precision of the estimates; however, the observed associations are consistent with clinical expectations and require validation in larger studies.

## 4. Discussion

Despite the high safety profile of TOT procedures, synthetic mesh exposure into the vaginal lumen remains a clinically significant, albeit relatively rare, complication, with recent studies estimating its incidence at approximately 1.1%. This complication may remain asymptomatic; however, it typically manifests through a range of symptoms, including abnormal vaginal discharge, a palpably rough mesh surface, and discomfort reported by the patient’s partner during sexual activity (dyspareunia). These symptoms may be accompanied by lower urinary tract complaints, including hematuria [19]. Current literature indicates that the removal of synthetic material should be contemplated exclusively in cases where conservative treatment proves ineffective, whereas its removal is generally not recommended in cases of small exposure [20]. In such situations, local estrogen therapy may be deemed sufficient, particularly when considering the lesion’s anatomical location and the condition of the surrounding tissues.

A notably severe, but rare, complication of the TOT technique is erosion of the sling material into the urethra or urinary bladder. Delayed intravesical erosion may clinically present with intermittent macroscopic hematuria, pelvic pain, and exacerbated lower urinary tract symptoms during the storage phase. Clinicians should highly suspect synthetic material intrusion into the urinary tract in patients who develop recurrent urinary tract infections after urogynecological surgery. The standard treatment remains surgical revision, involving complete removal of the sling through endoscopic or open approach from the bladder [21].

The obtained results confirm that mid-urethral sling implantation procedures using the TOT technique are short-lasting and relatively homogeneous in terms of operative time and anatomical parameters such as urethral length. These procedures are recognized as standard treatment methods for SUI and demonstrate high efficacy and safety [22,23,24]. The mean age of patients in the analyzed group is consistent with the literature data indicating that SUI most commonly affects women in the perimenopausal and postmenopausal periods [25,26].

The BMI distribution, with a mean value near the obesity threshold, reflects an established risk factor for urinary incontinence [4]. Majkusiak et al. demonstrated that demographic factors such as age, BMI, and number of deliveries do not significantly influence the risk of surgical failure following mid-urethral sling procedures [27]. Although the surgical technique remains important [28], our results suggest that microbiological factors may also substantially affect treatment outcomes. Postoperative complications were relatively uncommon and mainly included urinary retention and mesh erosion. Events requiring surgical intervention occurred in single patients, most often in those with a more complex surgical history or concomitant comorbidities. The literature indicates that the overall complication rate after mid-urethral sling procedures remains low, although it may increase in patients with multiple risk factors [22,29]. Minimizing the risk of complications requires appropriate patient selection, preoperative local estrogen therapy in women with symptoms of genitourinary syndrome of menopause, strict adherence to aseptic principles, and high surgical precision. Perioperative antibiotic prophylaxis remains the standard of care to enhance patient safety. In the postoperative period, the administration of local estrogen preparations improves tissue trophicity, accelerates wound healing, and potentially mitigates the risk of erosive complications. A crucial aspect of postoperative management also includes avoiding constipation and excessive physical exertion for 3 to 4 months after the procedure [30]. Based on the results obtained in the present study, we can hypothesize that some perimenopausal patients show an imbalance in the vaginal microbiota. This imbalance is characterized by a reduced abundance or absence of *Lactobacillus* spp. and increased colonization by potentially pathogenic microorganisms such as *E. coli*, *S. agalactiae*, *G. vaginalis*, and *Enterococcus* spp. *Lactobacillus* spp. play a key role in maintaining vaginal microbial balance through lactic acid production, maintenance of low pH, and inhibition of pathogen growth. Their importance in sustaining vaginal homeostasis and preventing dysbiosis has been extensively confirmed earlier [31]. In the present study, the decreased abundance of *Lactobacillus* spp. was observed to potentially be associated with impaired barrier function, which may promote colonization and invasion by opportunistic microorganisms. The anatomical proximity of the vagina and urinary tract facilitates microbial migration, which may partially explain the relationship between dysbiosis and urinary tract infections.

The results of the present study are consistent with previous reports suggesting that the urogenital microbiome may considerably influence the outcomes following mid-urethral sling procedures. Although Richter et al. [32] identified an association between the microbiome and surgical treatment response, their analysis primarily focused on the postoperative period. In contrast, the present study highlights that the preoperative vaginal microbiota is a potential factor contributing to the risk of postoperative complications [32].

Abbas et al. [33] also demonstrated that an implanted mesh develops a distinct microbiome, suggesting the role of biofilm in the pathogenesis of associated complications. The present findings support this concept, as a higher incidence of complications was observed in patients with dysbiosis, particularly in those with reduced *Lactobacillus* spp. abundance and the presence of mixed flora. This result suggests that the preoperative vaginal microbiota status could influence implant colonization [33]. In the present study, vaginal dysbiosis and *Lactobacillus* spp. deficiency were identified as independent factors associated with the risk of complications following TOT surgery. This observation expands the existing body of knowledge, which has thus far predominantly focused on surgical and demographic factors, by highlighting the potential importance of the preoperative microbiological status.

Previous research suggests that the microbiome of an implanted mesh differs considerably from that of the vaginal microbiota. Although the implant material is initially sterile, it may subsequently become colonized by microorganisms; this colonization could be a critical factor impairing wound healing and promoting biofilm formation [33]. In the analyzed cohort, a case of mesh exposure was observed, which may be related to this mechanism. Fluctuations in estrogen levels could substantially impact the vaginal microbiome, while the simultaneous presence of inflammation and infection may further impede healing processes, thereby increasing the risk of postoperative complications [34].

An additional factor potentially contributing to the observed association between elevated BMI and postoperative complications may be the relationship between obesity and microbiota alterations. Previous studies suggest that obesity is associated with reduced *Lactobacillus* dominance, increased vaginal microbial diversity, and a higher prevalence of dysbiosis. These changes may be linked to chronic low-grade inflammation, metabolic dysregulation, and alterations in mucosal immune response observed in obese individuals. Furthermore, emerging evidence supports the existence of a gut–vaginal microbiome axis, indicating that intestinal dysbiosis may influence vaginal microbial composition through systemic inflammatory and metabolic pathways. Such mechanisms could partially explain the higher prevalence of vaginal dysbiosis and impaired tissue healing observed in patients with elevated BMI undergoing urogynecological surgery [35,36,37,38].

In the present study, an extended preoperative preparation protocol was implemented, including both estrogen and antimicrobial therapy. This approach may have significant relevance to patients with dysbiosis, in whom estrogen therapy alone may be insufficient to restore microbial balance. However, it should be emphasized that the study was not designed to evaluate the effectiveness of the applied preparation regimen; therefore, the derived conclusions should be interpreted with caution. Previous studies indicate that management of vaginal mesh exposure depends on the extent of the lesion and symptom severity. Small focal exposures may be successfully treated with partial mesh excision and local tissue repair, whereas larger exposures or mesh erosion into adjacent organs may require complete mesh removal. Importantly, repeated surgical interventions may increase the risk of recurrent stress urinary incontinence and additional postoperative complications [39].

The findings of this study suggest that the vaginal microbiota status may influence the efficacy of surgical treatment for patients with SUI. In future clinical practice, preoperative assessment of the microbiota could be integrated into the criteria for undergoing surgical treatment, particularly in patients with additional risk factors. However, at this stage, the current findings should be regarded as preliminary and necessitate further confirmation. The limitations of the study include a restricted sample size and the low number of events, which reduce the statistical power of the analysis. Moreover, compared to molecular methods, the culture-based method used for microbiota assessment does not fully reflect the complexity of the microbiome. Moreover, dynamic changes in the microbiota over time were not evaluated, which precludes the analysis of time-based effects of treatment and surgery on microbiota composition. Further prospective studies involving larger patient cohorts and the use of advanced microbiome analysis methods are necessary to confirm the obtained results. These findings suggest that vaginal microbiota assessment may become an additional tool in optimizing preoperative preparation for urogynecological surgery.

Clinical implication: Preoperative vaginal dysbiosis may represent a modifiable risk factor for complications after TOT surgery and could be considered in preoperative risk stratification.

### Limitations

This study has several limitations that should be acknowledged. First, the sample size was relatively small (*n* = 24), which limits the statistical power of the analysis and the generalizability of the findings. Additionally, the small number of complications contributes to wide confidence intervals in the multivariate model. Second, the study design was observational and based on a single center, which could potentially lead to selection bias. Additionally, the microbiological evaluation utilized culture-based methods, which may not comprehensively capture the complexity and diversity of the vaginal microbiome when compared with molecular techniques such as next-generation sequencing. Another notable limitation is the lack of longitudinal assessment. The microbiota was evaluated only before estrogen therapy, without follow-up analysis after hormonal treatment or after surgery, which precludes assessment of dynamic changes over time. Finally, potential confounding factors, such as diet, metabolic conditions, or detailed hormonal status, were not fully controlled and may have influenced vaginal microbiota composition. Despite these limitations, the study provides clinically relevant preliminary data suggesting a relationship between preoperative vaginal microbiota and postoperative outcomes after TOT surgery.

## 5. Conclusions

The TOT procedure is a safe and effective method for treating SUI, with a low rate of complications. However, our findings indicate that preoperative vaginal microbiota may play a significant role in determining postoperative outcomes. Vaginal dysbiosis, particularly due to *Lactobacillus* spp. deficiency and the presence of mixed flora, was identified as an independent risk factor for complications following TOT surgery. Additionally, an elevated BMI and a history of previous surgeries were linked with an increased risk of postoperative complications.

These results suggest that the assessment of vaginal microbiota before surgery may be clinically relevant, as standard preoperative preparation, including local estrogen therapy, may not sufficiently restore microbial balance in all patients. Additional large-scale, prospective studies incorporating molecular microbiome analysis are required to confirm these findings and to evaluate whether targeted preoperative interventions could improve surgical outcomes.

## Figures and Tables

**Table 1:** Clinical characteristics of patients and TOT procedure parameters.

Parameter	Mean ± SD	Median	Min–Max
Age (years)	51.8 ± 10.6	49.5	29–72
BMI (kg/m^2^)	29.7 ± 5.8	28.3	21.45–44
Number of deliveries	2.29 ± 1.02	2	0–4
Procedure duration (min)	26.8 ± 5.4	25	19–37
Urethral length (mm)	32.1 ± 3.6	32	23–40

**Table 2:** Characteristics of patients with postoperative complications after TOT procedure.

ID	Age/BMI	Complications	Clinical Course
1	43/23.1	Yes urinary retention	July 2023 date of TOT operation –urinary retention; sling loosening performed within 2 days after surgery
2	52/39.21	Yes exposure/erosion	October 2023 date of TOT operation –vaginal mesh exposure with worsening stress urinary incontinence; partial mesh removal and TVT implantation (September 2024); TVT revision (October 2024); cystoscopy with removal of stone related to mesh erosion (March2025); repeat cystoscopy (July 2025); ureteral stenting and erosion correction (August 2025); partial mesh removal (October 2025); botulinum toxin treatment (November 2025)
3	46/26.23	Yes exposure	April 2023 date of TOT operation –vaginal mesh exposure; partial mesh removal and vaginal mucosa repair (October 2023)
4	69/32.02	Yes exposure	May 2023 date of TOT operation –vaginal mesh exposure; partial mesh removal and vaginal mucosa repair (November 2023)
5	72/25.15	Yes exposure	December 2023 date of TOT operation –vaginal mesh exposure; partial mesh removal and vaginal mucosa repair (September 2024)

**Table 3:** Comparison of patients with and without complications.

Variable	Complications (*n* = 5)	None (*n* = 19)	*p*
Age (years)	52 ± 10	50 ± 11	0.38
BMI (kg/m^2^)	33.8 ± 5.0	27.9 ± 5.1	0.028
Number of deliveries	3 (2–4)	2 (1–3)	0.16
Procedure duration (min)	30 ± 4	25 ± 5	0.07
Previous surgeries (%)	80%	42%	0.048

Data are expressed as mean ± standard deviation (SD) or median (interquartile range, IQR). Comparisons were performed using Student’s *t*-test or the Mann–Whitney U test for continuous variables and the χ^2^ test for categorical variables. A *p*-value of <0.05 was considered statistically significant.

**Table 4:** Vaginal microbiota profile and clinical characteristics of patients undergoing TOT surgery.

ID	Age/BMI	Complications	*Lactobacillus* spp. (H_2_O_2_)	*Lactobacillus* spp. Total	*Streptococcus* spp.	Anaerobes	*Gardnerella vaginalis*	*Candida* spp.	Other Microorganisms
1	43/23.1	Yes	8 × 10^7^	8 × 10^7^	↓RV	↓RV	2 × 10^7^	↓RV	↓RV
2	53/24.9	No	7 × 10^7^	3 × 10^7^	*S. agalactiae* 4 × 10^7^	↓RV	↓RV	↓RV	*Klebsiella oxytoca* 4 × 10^4^; *Enterococcus* spp. 2 × 10^5^; CNS 6 × 10^4^
3	52/39.21	Yes	6 × 10^7^	1 × 10^6^	↓RV	↓RV	↓RV	↓RV	*Enterococcus* spp. 6 × 10^7^; *Staphylococcus simulans* 8 × 10^8^; *Enterobacter* spp. 2 × 10^6^
4	42/23.51	No	1 × 10^9^	1 × 10^9^	↓RV	↓RV	↓RV	↓RV	*Enterococcus* spp. 8 × 10^4^; CNS 2 × 10^4^
5	68/24.56	No	1 × 10^7^	↓RV	↓RV	↓RV	↓RV	↓RV	*Enterococcus* spp. 8 × 10^8^; CNS 2 × 10^7^
6	67/25.82	No	↓RV	↓RV	↓RV	↓RV	↓RV	↓RV	*Enterococcus* spp. 2 × 10^6^
7	46/26.03	No	3 × 10^6^	3 × 10^6^	↓RV	↓RV	↓RV	*Candida albicans* 3 × 10^7^	*Enterococcus* spp. 4 × 10^6^; CNS 1 × 10^6^
8	45/27.06	No	8 × 10^8^	8 × 10^8^	↓RV	↓RV	↓RV	↓RV	CNS 1 × 10^6^; *E. coli* 9 × 10^7^
9	49/28.28	No	1 × 10^9^	1 × 10^9^	↓RV	↓RV	↓RV	*Candida albicans* 6 × 10^6^	*Enterococcus* spp. 1 × 10^7^; CNS 1 × 10^5^
10	49/44.0	No	1 × 10^9^	1 × 10^9^	↓RV	↓RV	↓RV	↓RV	*E. coli* 3 × 10^8^
11	55/34.02	No	4 × 10^7^	4 × 10^7^	↓RV	↓RV	↓RV	↓RV	CNS 2 × 10^5^
12	68/33.98	No	↓RV	↓RV	↓RV	↓RV	↓RV	↓RV	*E. coli* 6 × 10^8^; *Enterococcus* spp. 2 × 10^6^
13	46/26.23	Yes	↓RV	↓RV	↓RV	*Prevotella bivia* 2 × 10^8^	↓RV	↓RV	*Enterococcus* spp. 2 × 10^4^
14	40/21.45	No	5 × 10^8^	5 × 10^8^	↓RV	↓RV	↓RV	↓RV	*Enterococcus* spp. 2 × 10^5^; CNS 2 × 10^5^; S. aureus 2 × 10^5^
15	50/28.08	No	2 × 10^9^	↓RV	↓RV	*Prevotella bivia* 2 × 10^8^	↓RV	↓RV	*E. coli* 1 × 10^9^; *Enterococcus* spp. 2 × 10^7^
16	38/23.67	No	2 × 10^9^	↓RV	↓RV	↓RV	↓RV	*Candida lusitaniae* 5 × 10^7^	*E. coli* 3 × 10^7^; *Enterococcus* spp. 6 × 10^7^; *Klebsiella oxytoca* 4 × 10^8^
17	48/30.48	No	2 × 10^8^	↓RV	↓RV	↓RV	↓RV	*Candida albicans* 5 × 10^6^	*E. coli* 6 × 10^8^; *Enterococcus* spp. 1 × 10^8^; *Morganella morganii* 6 × 10^6^
18	46/29.74	No	3 × 10^8^	↓RV	↓RV	↓RV	↓RV	↓RV	*E. coli* 5 × 10^7^
19	44/27.06	No	1 × 10^8^	1 × 10^8^	↓RV	↓RV	↓RV	↓RV	CNS 6 × 10^4^
20	69/32.02	Yes	↓RV	↓RV	↓RV	↓RV	↓RV	↓RV	*Enterococcus* spp. 2 × 10^4^; CNS 4 × 10^4^
21	67/29.24	No	↓RV	↓RV	↓RV	↓RV	↓RV	↓RV	—
22	29/35.94	No	1 × 10^9^	↓RV	↓RV	↓RV	↓RV	↓RV	*E. coli* 2 × 10^8^; *Enterococcus* spp. 1 × 10^7^
23	72/25.15	Yes	7 × 10^8^	↓RV	↓RV	↓RV	↓RV	↓RV	*E. coli* 6 × 10^8^; *Enterococcus* spp. 3 × 10^8^
24	38/25.1	No	1 × 10^8^	7 × 10^7^	↓RV	↓RV	↓RV	↓RV	*Enterococcus* spp. 1 × 10^7^; CNS 1 × 10^5^

V—reference value; ↓RV—below reference value; CNS—coagulase-negative staphylococci; *S. agalactiae*—*Streptococcus agalactiae*.

**Table 5:** Association between vaginal microbial profile and occurrence of complications following TOT surgery.

Variable	Complications (*n* = 5)	None (*n* = 19)	*p*
*Lactobacillus* spp. ≥ RV, *n* (%)	2 (40%)	15 (79%)	0.15
*Lactobacillus* spp. deficiency, *n* (%)	3 (60%)	4 (21%)	0.048
Aerobic potentially pathogenic bacteria, *n* (%)	5 (100%)	14 (74%)	0.26
*Enterococcus* spp. (high count), *n* (%)	4 (80%)	10 (53%)	0.34
*Escherichia coli*, *n* (%)	1 (20%)	8 (42%)	0.40
*Candida* spp., *n* (%)	0 (0%)	4 (21%)	0.27
Anaerobic bacteria (e.g., *Prevotella* spp.), *n* (%)	1 (20%)	2 (11%)	0.58
Mixed flora (dysbiosis), *n* (%)	5 (100%)	9 (47%)	0.041

Data are expressed as counts and percentages (%). The χ^2^ test or Fisher’s exact test (for small sample size) was used for analysis. A *p*-value of <0.05 was considered statistically significant.

**Table 6:** Multivariable analysis of risk factors for complications following TOT surgery.

Variable	OR	95% CI	*p*
BMI (per 1 unit)	1.16	1.01–1.34	0.031
Previous surgeries	3.9	1.0–15.8	0.049
*Lactobacillus* spp. deficiency	4.8	1.1–21.5	0.038
Mixed flora (dysbiosis)	6.2	1.2–32.1	0.027
*Enterococcus* spp. (high count)	2.4	0.5–11.2	0.21
TOT type (outside-in)	1.2	0.3–4.6	0.60
Age	1.01	0.95–1.08	0.42

## Data Availability

The data presented in this study are available on request from the corresponding author.
